# Supplementary material for: Liver-Specific Nanoparticle-Mediated Delivery and MMP-Triggered Release of Veratridine to Effectively Target Metastatic Colorectal Cancer
Source: Cancers (Basel). 2025 Oct 8;17(19):3253. doi: 10.3390/cancers17193253 (PMC12524202; doi:10.3390/cancers17193253)
Supplement: Supplementary file 1 [file cancers-17-03253-s001.zip › cancers-3849727-supplementary.pdf]

Supplementary Materials

Article

Liver-Specific Nanoparticle Delivery and MMP-Triggered Release of Veratridine to Effectively Target Metastatic Colorectal Cancer

Mahadi Hasan [1,†], Morgan Eikanger [2,†], Sanam Sane [2], Krishantha S. K. Wijewardhane [1], John L. Slunecka [2], Jessica Freeling [2], Khosrow Rezvani [2,*] and Grigoriy Sereda [1,*]

[1] Department of Chemistry, University of South Dakota, 414 E Clark Street, Vermillion, SD 57069, USA; mahadi.hasan01@coyotes.usd.edu (M.H.); sajith.wijewardhane@usd.edu (K.S.K.W.)

[2] Division of Biomedical and Translational Sciences, Sanford School of Medicine, University of South Dakota, 414 E Clark Street, Vermillion, SD 57069, USA; morgan.eikanger@coyotes.usd.edu (M.E.); sanam.sane@usd.edu (S.S.); slune008@umn.edu (J.S.); jessica.freeling@usd.edu (J.F.)

* Correspondence: khosrow.rezvani@usd.edu (K.R.); grigoriy.sereda@usd.edu (G.S.)

† These authors contributed equally to this work.

Table S1. Used antibodies.

Name	Manufacturer and Catalog number	Dilution	Catalog number
IRDye 800CW Goat anti-Rabbit IgG (H+L),	LI-COR Corporate	1:3000	827-08365
925-68020 IRDye® 680LT Goat anti-Mouse IgG (H + L)	LI-COR Corporate	1:3000	925-68020
Human BD Fc Block	BD Pharmingen	1 μl/10^6 cells	564220
Ms IgG2b Kpa PE	BD Pharmingen	1 μl/10^6 cells	555743
PE Mouse Anti-Human CD44	BD Pharmingen	1 μl/10^6 cells	561858
APC Mouse Anti-Human CD133	BD Pharmingen	1 μl/10^6 cells	566597
APC Mouse IgG1 k Isotype Control	BD Pharmingen	1 μl/10^6 cells	554681
Alexa Flour 647 Rat IgG2b, k Isotype Control	BD Pharmingen	1 μl/10^6 cells	557691
Rabbit polyclonal anti-UBXN2A against #C-IQRLQKTASFRELS peptide located in the c-terminus of human UBXN2A (#NM_181713)	Pacific Immunology Corp	1:1000	-
8762S Bid (3C5) Mouse mAb	Cell Signaling Technology	1:500 for WBs	
Caspase-8 (1C12) Mouse mAb	Cell Signaling Technology	1:500 for WBs	9746S
Anti-β tubulin	Millipore Sigma	1:100 for WBs	T8328

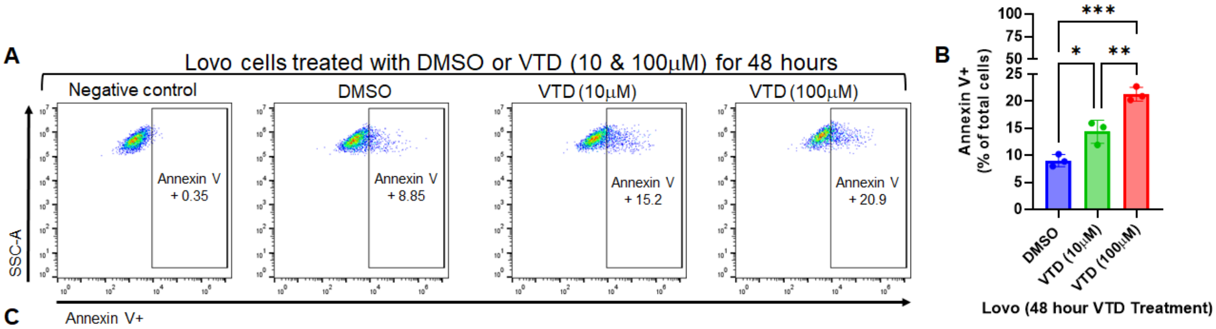

C

	A	B	C	D	E	F	G	H	I	J	K	L	M	N
1	Pos	Pos	Neg	Neg	BLANK	BLANK	Bad	Bax	Bcl-2	Bcl-w	BID	BIM	Caspase 3	Caspase 8
2	Pos	Pos	Neg	Neg	BLANK	BLANK	Bad	Bax	Bcl-2	Bcl-w	BID	BIM	Caspase 3	Caspase 8
3	CD40	CD40L	cIAP-2	cytoC	DR6	Fas	FasL	BLANK	HSP27	HSP60	HSP70	HTRA	IGF-I	IGF-II
4	CD40	CD40L	cIAP-2	cytoC	DR6	Fas	FasL	BLANK	HSP27	HSP60	HSP70	HTRA	IGF-I	IGF-II
5	IGFBP-1	IGFBP-2	IGFBP-3	IGFBP-4	IGFBP-5	IGFBP-6	IGF-1sR	Livin	p21	p27	p53	SMAC	Survivin	sTNF-R1
6	IGFBP-1	IGFBP-2	IGFBP-3	IGFBP-4	IGFBP-5	IGFBP-6	IGF-1sR	Livin	p21	p27	p53	SMAC	Survivin	sTNF-R1
7	sTNF-R2	TNF-α	TNF-β	TRAILR-1	TRAILR-2	TRAILR-3	TRAILR-4	XIAP	BLANK	BLANK	Neg	Neg	Neg	Pos
8	sTNF-R2	TNF-α	TNF-β	TRAILR-1	TRAILR-2	TRAILR-3	TRAILR-4	XIAP	BLANK	BLANK	Neg	Neg	Neg	Pos

Figure S1: VTD induces both intrinsic and extrinsic apoptotic pathways in colon cancer cells. We have already shown that VTD can induce the apoptosis pathway. To further understand the apoptotic cascades activated by VTD, we first treated LoVo colon cancer cells with VTD (10μM and 100μM) for 48 hours. The elevated level of Annexin V apoptotic marker revealed that LoVo cells potently respond to the cytotoxicity impact of VTD in a dose-dependent manner (**A-B**). Next, LoVo cells were incubated for 72 hours with VTD (100μM) to maximize the number of apoptotic cells before collection. Cell lysates were subjected to a human apoptosis antibody array (**C**). The array revealed 32 out of 43 apoptotic-associated proteins increased by greater than or equal to 25% in response to VTD (**C**). Confirmation of these changes in primary and metastatic colorectal cancer cells in the presence of VTD is an ongoing project in our group.

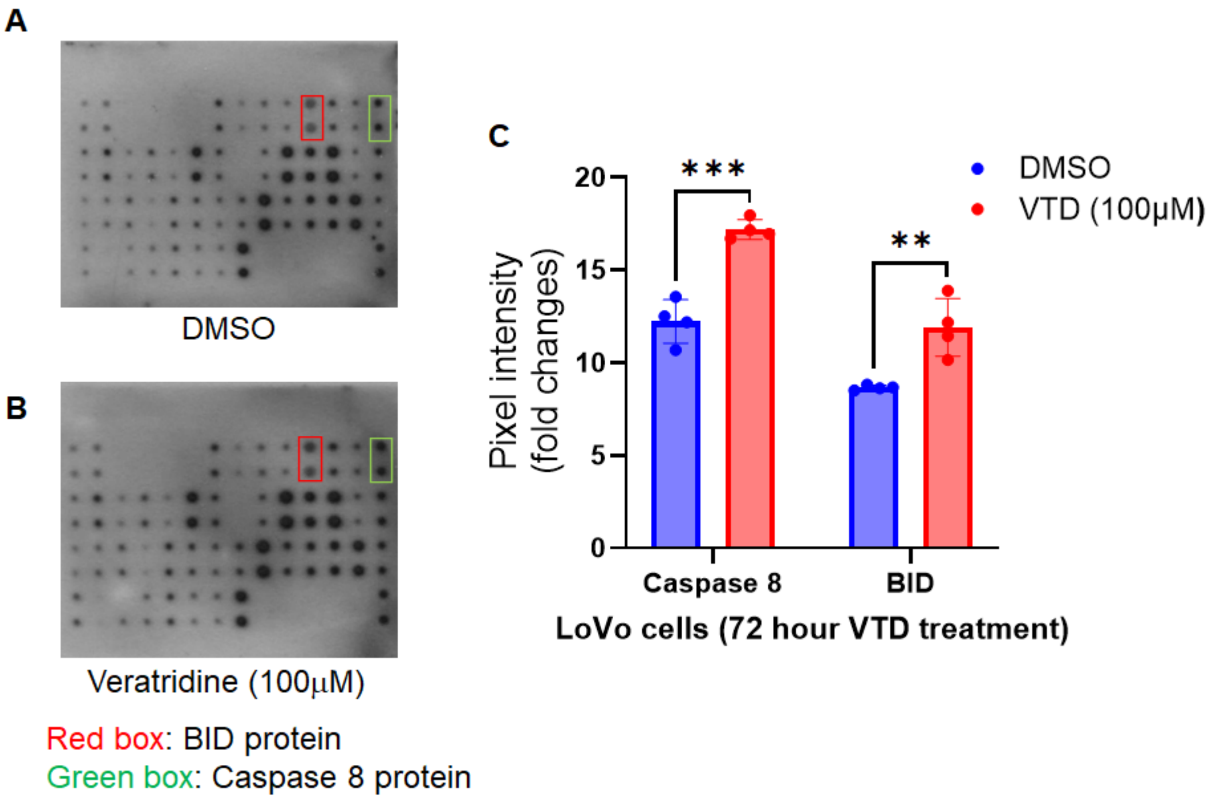

Figure S2: VTD increases proteins essential for the induction and maintenance of the extrinsic apoptotic cascade in CRC. Quantitation of signals recorded on X-ray film after ECL revealed a significant elevation of caspase 8 and BID proteins after LoVo cells were treated with VTD (100µM) for 72 hours (**A-C**). This apoptotic screening assay revealed that VTD can trigger the formation of active caspase 8, which consequently cleaves BID within a native complex on the mitochondrial membrane. Activated cleaved BID on the mitochondria is an essential mediator for caspase-8-induced cytochrome c release, resulting in apoptotic cell death (n=4 in duplicate, $^{**}P<0.01$, $^{***}P<0.001$, mean±SD).

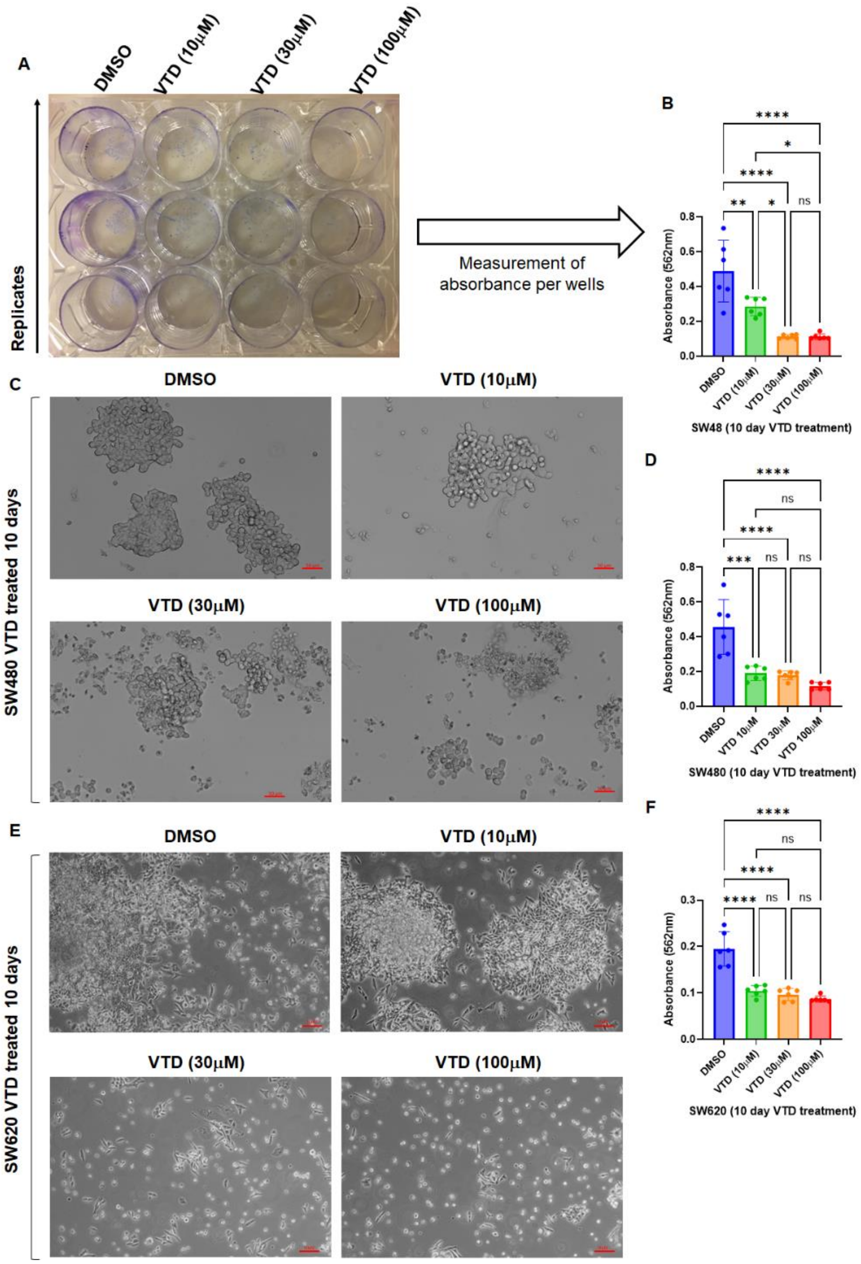

Figure S3: Overexpression or induction of UBXN2A inhibits cell proliferation without affecting normal colon cells. The SW48, SW480, and SW620 cells were plated on 6-well plates at 10,000-50,000 cells/well for each experiment, respectively. Based on the plating efficiency of each cell line, the number of cells plated per well was adjusted. Twenty-four hours after cell adhering, cells were treated with DMSO or VTD (100μM) for 10 days, a sub-chronic treatment. The cell viability was measured using the crystal violet assay, followed by the O.D. of the crystal violet staining at 590 nm (**A**). The control groups plated with different cell numbers confirmed that the generated absorbance numbers are directly proportional to cell biomass (data not shown). All measurements were performed in 2 replicates (3 wells per treatment). Crystal assay results for all cells (**B, D, F**) alongside SW480 (**C**) and SW620 (**E**) morphology revealed that VTD can significantly decrease cell viability and number of both primary and metastatic colon cancer cells in a dose-dependent manner. (n=6, duplicate, *P<0.05, **P<0.01, ***P<0.001, ****p<0.0001, mean±SD).

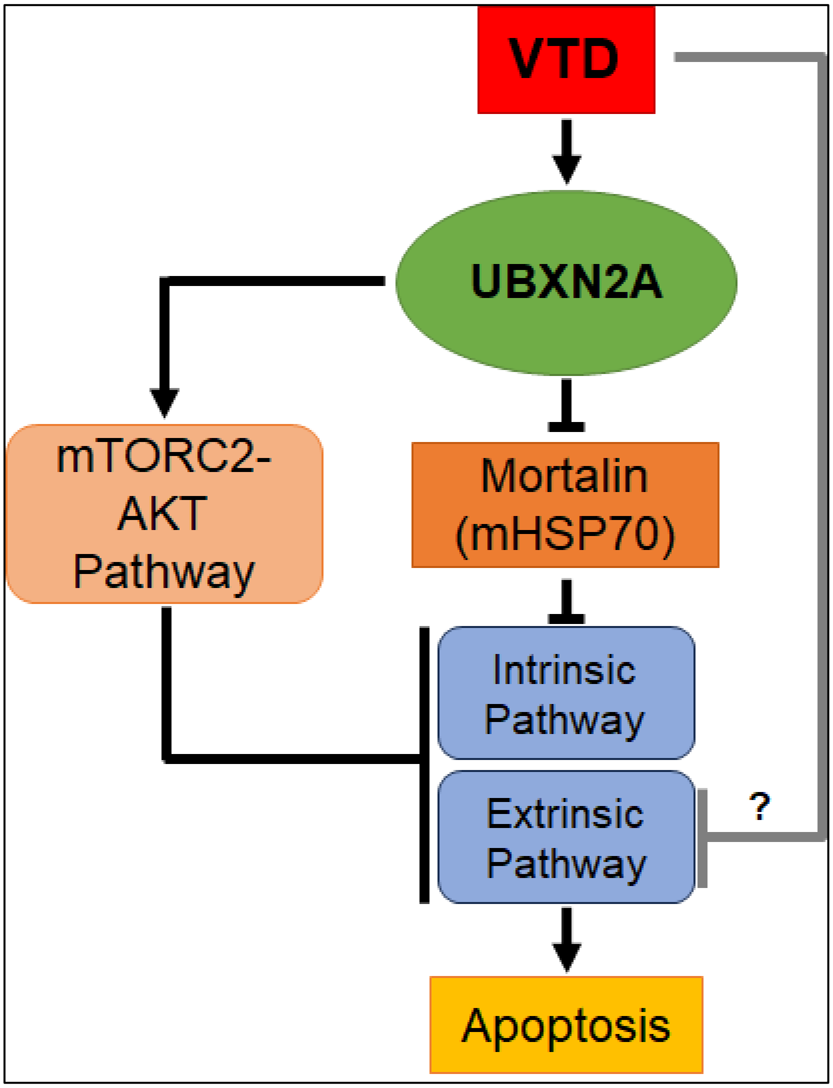

Figure S4: VTD initiates the Apoptotic Cascade by two mechanisms. The schematic diagram shows that the VTD-UBXN2A axis uses a dual targeting function of both the apoptotic and AKT pathways to generate a progressive apoptotic cascade in cancer cells.

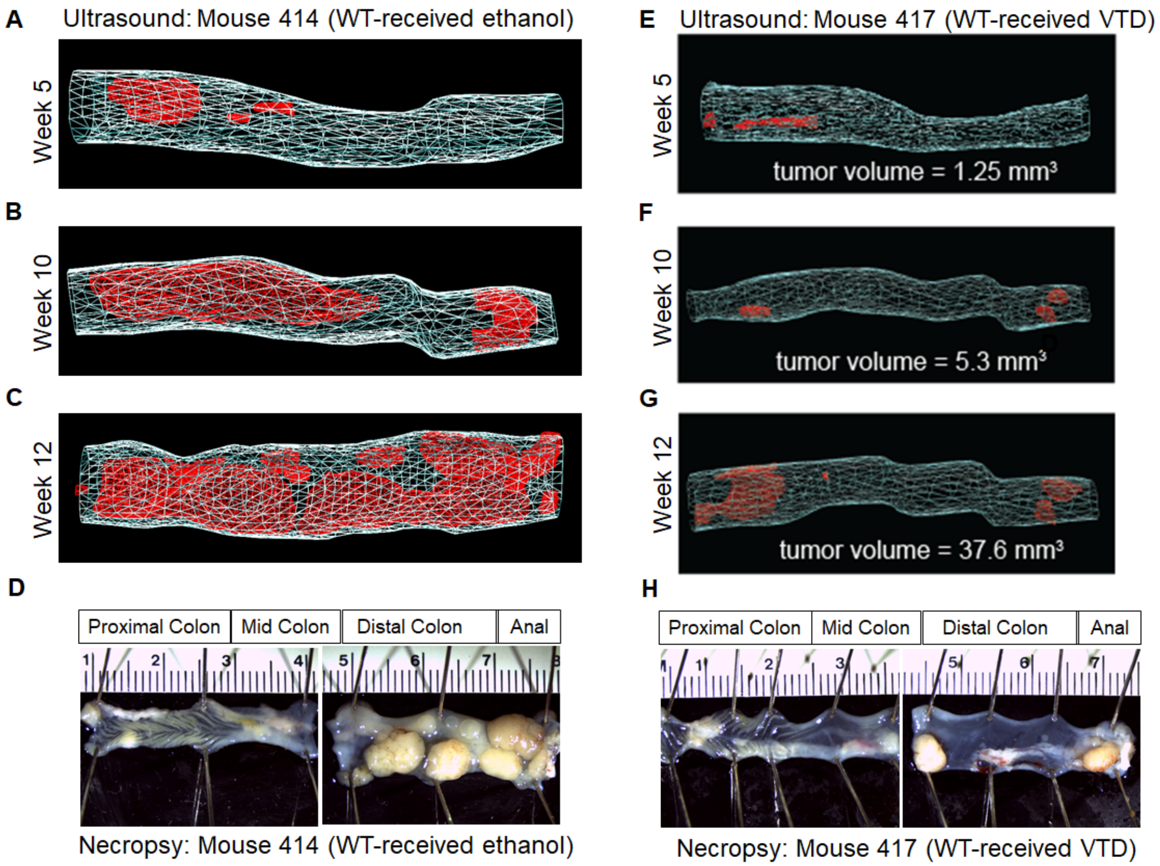

Figure S5: VTD reduces tumor growth in a murine mouse model of colorectal cancer. Panels **A-D** (WT-C57BL/6 received ethanol) and **E-F** (WT C57BL/6-received 0.1 mg/kg VTD) represent ultrasound and post-construction of developing tumors in mice that received AOM/DSS. The volume of 3D reconstructed tumors in pre-, post-, and terminal stages was measured. Mice were terminated, and developed tumors were imaged by a Lecia microscope. The ethanol and VTD-treated results shown in this figure were originally used in another methodology development article 2016 published by our group [42]. However, the current results shown in Supplementary Figure 5 show the treatment groups that support the unpublished UBXN2A (+/-) results and statistical analysis presented in the main Figure 2. The authors obtained written permission from Alexi Starr, the Editorial Assistant of Molecular Therapy Family of Journals (American Society of Gene and Cell Therapy, asgct.org) for re-presenting the results in Supplementary Figure S5.

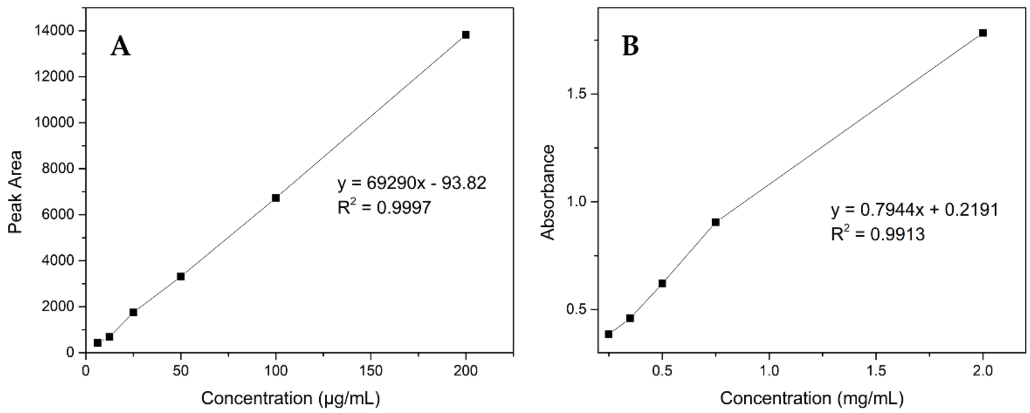

Figure S6. Calibration curves for determining VTD loading and release using HPLC, and for calculating unbound casein with a plate reader. The amount of VTD loaded and released from MSN-COOH/VTD/CAS was determined. The standard curve was created by plotting known concentrations of VTD (200, 100, 50, 25, 12.5, and 6.25 µg/mL) against the peak area obtained from HPLC. The HPLC parameters were as follows: flow rate 0.35 mL/min, pump pressure 800-900 psi, temperature 20°C, and VTD's retention time was 17.5 to 18.5 minutes at a wavelength of 220 nm **(A)**. The amount of casein chemically conjugated into VTD-loaded particles during the formation of MSN-COOH/VTD/CAS was measured by plotting several known concentrations of analytical grade blocker casein (2, 0.75, 0.5, 0.35, and 0.25 mg/mL) against absorption values from the UV microplate reader to establish this calibration curve **(B)**. The samples' absorbance was measured at 562 nm.

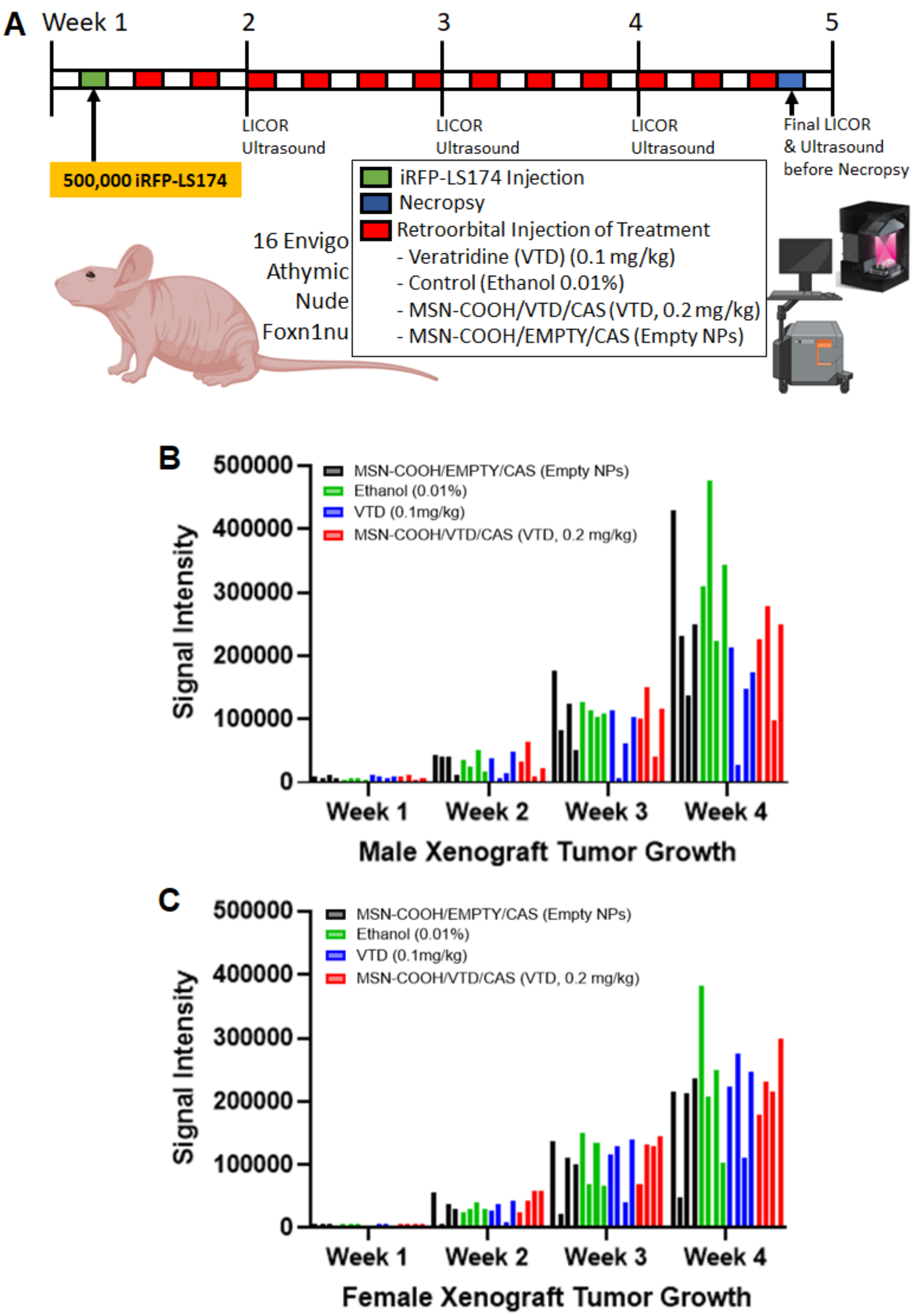

Figure S7: Xenograft experiment with VTD and MSN-COOH/VTD/CAS. The timeline shows the implantation of iRFP-LS174 cells, treatment every other day, and necropsy **(A)**. Mice were imaged weekly using LICOR and Leica ultrasound. Tumor growth in males **(B)** and females **(C)** was tracked by signal intensity from the iRFP tag on the LS174 cells over four weeks.

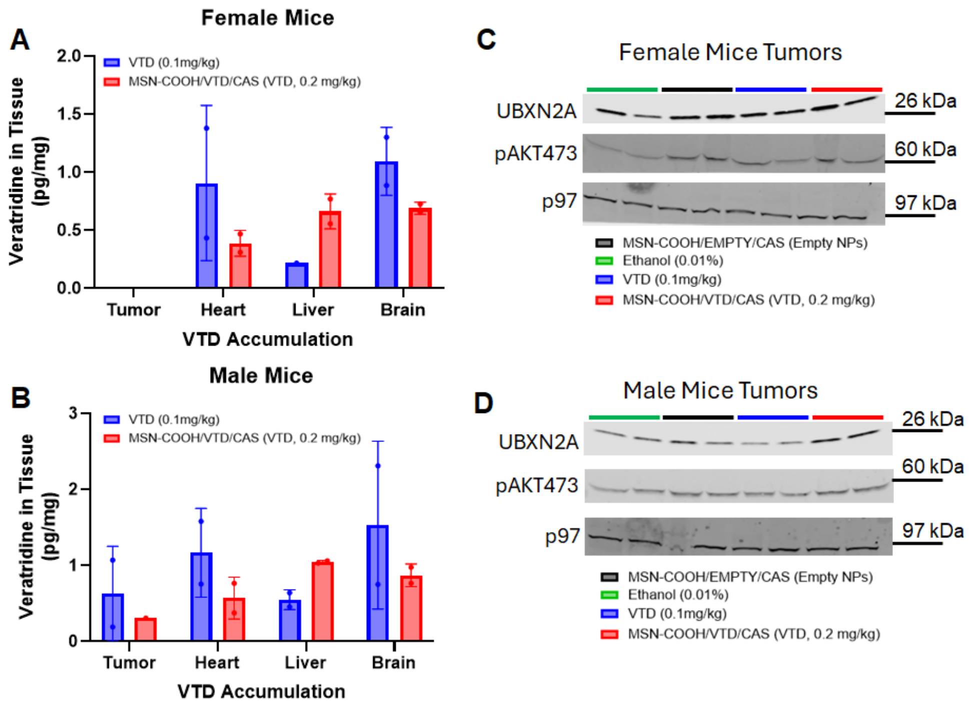

Figure S8: Outcome of treatment with Veratridine (VTD) and MSN-COOH/VTD/CAS. At the endpoint, the tumors, hearts, livers, brains, and sera were collected from each mouse. The tumors on the right and left flanks were collected and cut in half. Half of the tumor was sent to the toxicology core at the University of Utah where they used mass spectrometry to measure Veratridine accumulation. The heart and brain tissues revealed less accumulation of VTD in mice treated with MSN-COOH/VTD/CAS compared to pure VTD, indicating the nanoparticle effectively retains encapsulated VTD in non-cancerous tissues. Interestingly, we observed an accumulation of VTD in the liver tissues of mice that received MSN-COOH/VTD/CAS, which is likely due to the liver's predominant trapping of nanoparticles due to their size and subsequent metabolism of VTD. Female mice have no detectable traces of VTD in tumor tissues, which could be due to the rapid metabolism of VTD in female liver tissue or a technical error during mass spectrometry analysis (**Panel A**). In male mice, there is an accumulation of VTD in mice treated with pure VTD and MSN-COOH/VTD/CAS nanoparticle (**Panel B**). The other half of the tumor was used for WB experiments, and p97 protein (Santa Cruz) was used for loading control. There were no meaningful changes in UBXN2A and pAKT473 protein in female mice (**Panel C**). Results revealed there is a remarkable elevation of UBXN2A in male mice treated with MSN-COOH/VTD/CAS compared to pure VTD (**Panel D**). The absence of elevated UBXN2A levels in the mice treated with pure VTD may be due to the significant necrotic tissue in the tumor after 4 weeks of treatment, as previously reported by our group [42]. Expectedly, we observed a partial reduction of pAKT473 protein in male mice treated with MSN-COOH/VTD/CAS after normalization. The experiments presented in this figure represent n=2 samples; therefore, no statistical analysis was conducted.

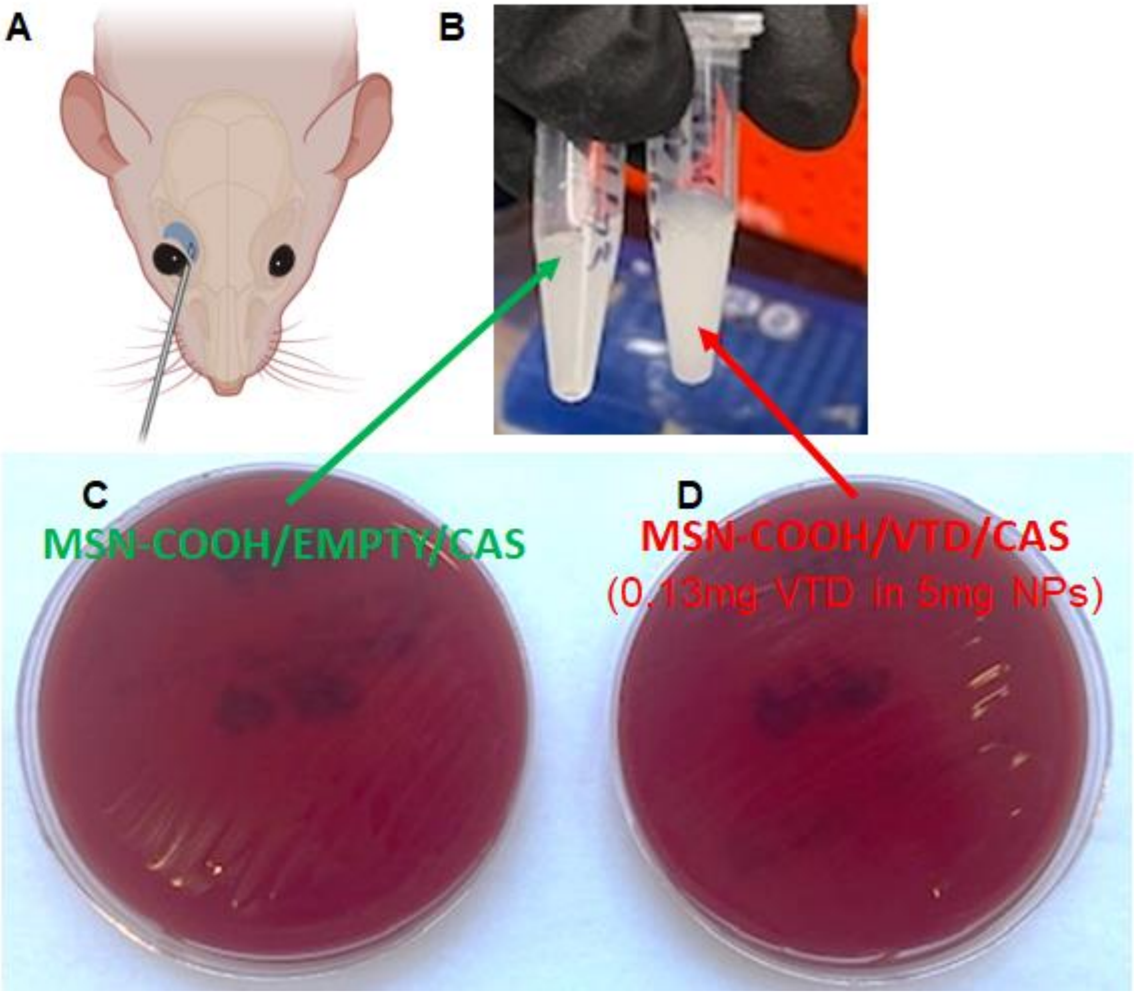

Figure S9: Sterile homogeneous suspension of MSN-COOH/VTD/CAS and MSN-COOH/EMPTY/CAS nanoparticles. To evaluate the sterility of the homogeneous nanoparticle suspensions before retroorbital injection **(A)**, a 50μL aliquot of either the NP-VTD or NP-Empty suspension (B) was aseptically spread onto individual 5% sheep blood agar plates (Hardy Diagnostics, Santa Maria, California, USA). Plates were then incubated at 37 °C for 24 hours under aerobic conditions. Sterility was confirmed by the complete absence of visible microbial growth on the agar surface after incubation **(C-D)**.

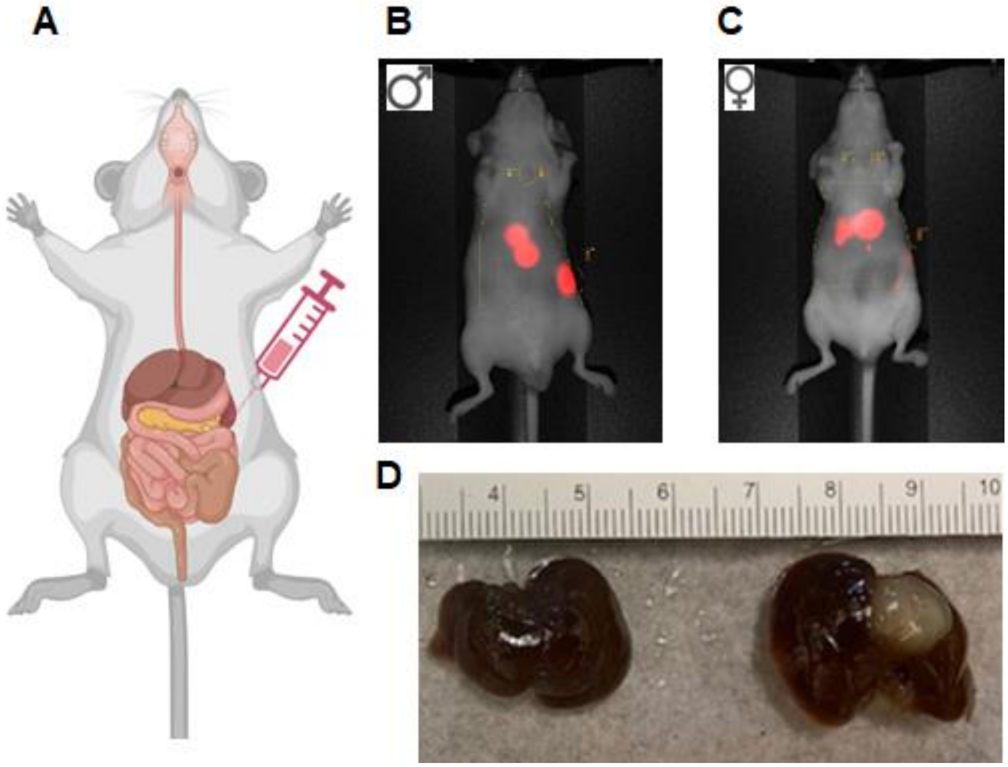

Figure S10: Generation of a stable hepatic metastasis mouse model of murine colorectal cancer by microsurgical orthotopic injection.

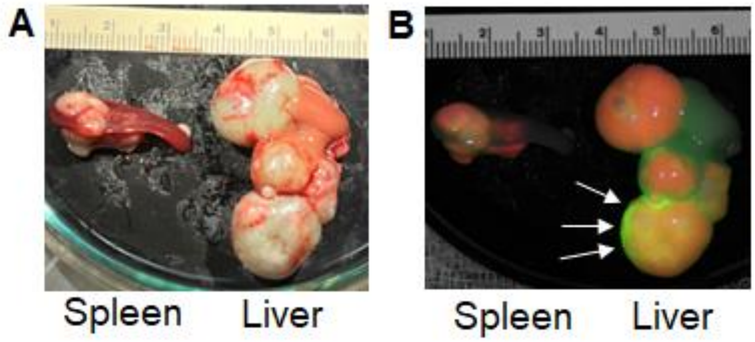

Figure S11: Mesoporous silica nanoparticles are enriched in the liver and exhibit affinity for the tumor. IRDye 800CW NHS Ester (green) was conjugated to casein-coated nanoparticles. The resected spleen and liver were imaged using a Leica microscope **(A)** and LI-COR Odyssey **(B)**. There is an enrichment of green fluorescent signal around the tumor, indicating the affinity of the MSNs for the metastatic tumor site in the liver.

42. Freeling, J.L. and K. Rezvani, *Assessment of murine colorectal cancer by micro-ultrasound using three dimensional reconstruction and non-linear contrast imaging*. Molecular Therapy — Methods & Clinical Development, 2016. **5**: p. 16070.
